# Omalizumab: clinical use for the treatment of an adolescent with difficult asthma

**DOI:** 10.1186/2045-7022-3-S1-P29

**Published:** 2013-05-03

**Authors:** Giovanna De Castro, Taulant Melengu, Maria Palma Carbone, Annalisa Di Coste, Gabriella Giancane, Paola Pansa, Valentina De Vittori, Marzia Duse

**Affiliations:** 1 Università La Sapienza, Policlinico Umberto 1, Servizio di Allergologia ed Immunologia Pediatrica, Italy

## 

We describe the case of a 14 year old girl, followed by our Pediatric Allergology and Immunology Service for persistent rhinitis and asthma. The child suffered from asthma since the age of three years with a worsening of symptoms during the winter months. At 4 years of life, she was hospitalized for the first severe asthma episode. The SPT were positive for dust mites, *Alternaria*, pollens of grasses, Cypress, Birch, Plane, epithelium of dog and cat, fish and soy. She started a fish and soy free diet and therapy with ICS (50 mcg daily for 2), antihistamine and antileukotriene. At six years of life she performed spirometry, that showed moderate airflow obstruction (FEV1 75.8%) and significant dilatation after salbutamol (+16%). For persistence of asthma despite the therapy, at the age of 8 years she added LABA to the ICS (25/125 mcg daily for 2), with significant improvement for the next two years; spirometry normalized (FEV1 88.2%). When ten, she started with almost daily wheezing that required the use of OCS; spirometry showed severe bronchial obstruction and restriction (FEV1 58.8%). Chest X-ray was performed, showing peribronchial infiltration and air trapping; ph-metry, sweat test and Mantoux were negative; HRCT showed areas of thickening with appearance "ground glass" air trapping and bronchiectasis predominantly in the upper lobes. Immunological and autoimmune evaluation were negative. Monitoring with the Peak Flow Meter showed the persistence of frequent and severe symptoms; FEV1 was 48.5% with expansion of 28.6% after salbutamol. The girl was considered a candidate for therapy with omalizumab and this was started in Autumn 2011 (575 mg/2 weeks) with significant improvement (FEV1 100.5%). To date she is still treated with omalizumab, plus therapy with CSI + LABA, antileukotriene and antihistamine, with good control of asthma. On the basis of our experience, the use of omalizumab is an effective treatment for asthma resistant to common therapies, to reduce bronchial reactivity, symptoms and use of OCS.

